# Prevalence of the emerging novel Alongshan virus infection in sheep and cattle in Inner Mongolia, northeastern China

**DOI:** 10.1186/s13071-019-3707-1

**Published:** 2019-09-12

**Authors:** Ze-Dong Wang, Wei Wang, Ni-Na Wang, Kai Qiu, Xu Zhang, Gegen Tana, Quan Liu, Xing-Quan Zhu

**Affiliations:** 1 State Key Laboratory of Veterinary Etiological Biology, Lanzhou Veterinary Research Institute, Chinese Academy of Agricultural Sciences, Lanzhou, 730046 Gansu People’s Republic of China; 2 School of Life Sciences and Engineering, Foshan University, Foshan, 528225 Guangdong People’s Republic of China; 3 Military Veterinary Institute, Academy of Military Medical Sciences, Key Laboratory of Jilin Province for Zoonosis Prevention and Control, Changchun, 130122 Jilin People’s Republic of China; 4 Center for Animal Disease Control and Prevention of Hulunbuir, Hulunbuir, 021000 Inner Mongolia Autonomous Region People’s Republic of China

**Keywords:** Alongshan virus, Enzyme-linked immunosorbent assay, Viral neutralizing antibodies, Quantitative real-time RT-PCR, Northeastern China

## Abstract

**Background:**

Alongshan virus (ALSV) is a novel discovered segmented flavivirus associated with human febrile illness in northeastern China. *Ixodes persulcatus* is considered as a candidate vector of ALSV in the endemic regions. However, the role of domesticated animals in the circulation and transmission of ALSV have not been investigated. To evaluate the prevalence of ALSV infections in domesticated animals, viral RNA and viral specific antibodies were detected in sheep and cattle in Hulunbuir of northeastern Inner Mongolia. The findings contribute to the understanding of the ecology and transmission of ALSV among different natural hosts.

**Methods:**

A total of 480 animal serum samples were collected in Hulunbuir of northeastern China in May, 2017. Viral specific antibodies were tested by indirect enzyme-linked immunosorbent assay (ELISA) with a purified *E. coli* recombinant capsid protein (VP2) of ALSV (strain H3) and further detected by viral neutralization test (VNT). RNA in serum samples were extracted and detected for ALSV sequence by quantitative real-time RT-PCR. ALSV RNA positive samples were used for virus isolation.

**Results:**

ALSV-specific antibodies were detected in 9.2% (22/240) of examined sheep and 4.6% (11/240) of examined cattle by ELISA, while lower serological positivity with 4.2% (10/240) for sheep and 1.7% (4/240) for cattle was confirmed by VNT. In contrast, the prevalence of ALSV RNA was much higher, ranging from 26.3% (63/240) in sheep to 27.5% (66/240) in cattle. The partial S1 (NS5-like) and S3 (NS3-like) segments of ALSVs in sheep and cattle shared high identities of more than 98% to the human and tick isolates in the studied regions.

**Conclusions:**

These results suggest that the natural infection of ALSV can be found in sheep and cattle in the endemic regions.

## Background

Alongshan virus (ALSV) is a newly discovered pathogenic virus of the segmented *Flavivirus*, Jingmenvirus, in the family *Flaviviridae* in northeastern China [1]. A recent study also detected ALSV in *Ixodes ricinus* ticks in southeastern Finland [2]. Jingmenviruses are highly diverse and are distributed in China, Brazil, Uganda and the USA [3–5]. They are evolutionarily related to the conventional flaviviruses that are capable of infecting a wide range of animal hosts [5]. All Jingmenviruses comprise four or five segments, two of which are related to the nonstructural protein genes (NS5 and NS2b-NS3) of the genus *Flavivirus*, while the remaining segments encode structural proteins that are unique to these viruses [1–5].

ALSV infection has been reported in farmers living in hilly, wooded or mountainous areas in Inner Mongolia and Heilongjiang provinces in the northeastern regions of China. The virus epidemic season starts from May and proceeds through July [1]*. Ixodes persulcatus* is considered as a candidate vector of ALSV, as ALSV RNA was detected in *I. persulcatus* that were collected from a field where patients were bitten, with a prevalence of 6.5% in Hulunbuir, Inner Mongolia [1]. However, the vertebrate hosts of the virus have not been investigated, and the role of domesticated animals in the circulation and transmission of ALSV needs to be further clarified. In China, most cases of ALSV infection are found in Inner Mongolia, especially in Hulunbuir [1]. To evaluate the prevalence of ALSV infection in domesticated animals, an epidemiological study was conducted in Hulunbuir of northeastern Inner Mongolia. We detected the prevalence of viral RNA and viral specific antibodies in sheep and cattle, and these findings would contribute to the understanding of the ecology and transmission of ALSV among different vertebrates.

## Methods

### Sample collection

**Fig. 1:**
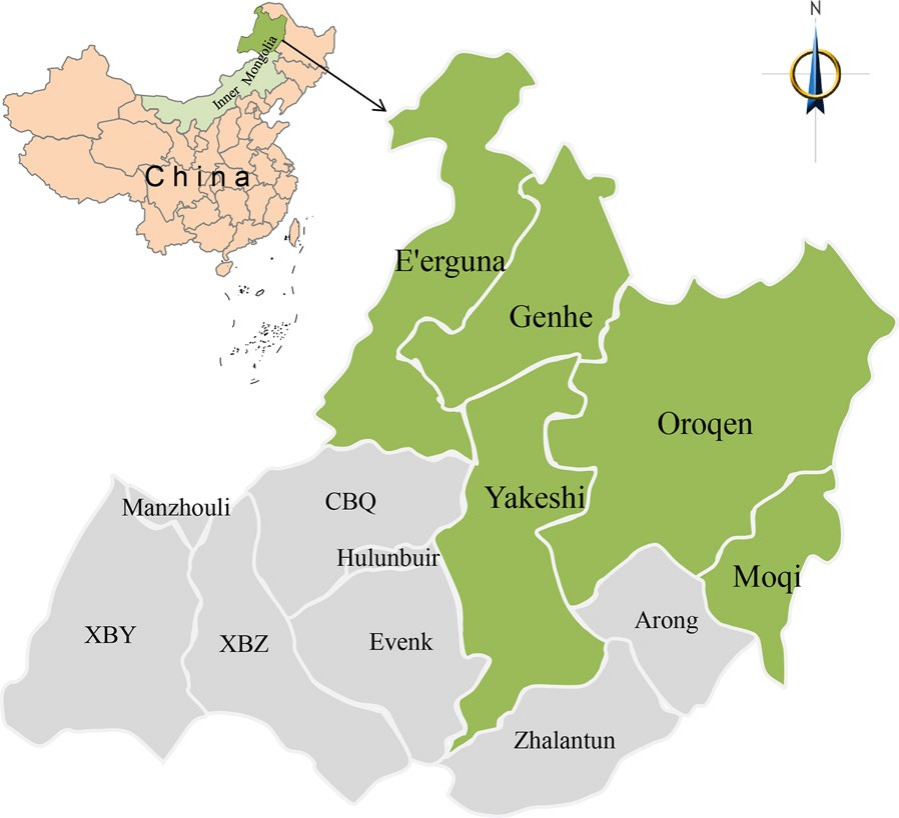
Sampling locations of sheep and cattle for the present survey in Hulunbuir, northeastern Inner Mongolia of China. Green shadowed areas indicate the sampling locations. *Abbreviations*: CBQ, Chen Barag Qi; XBZ, Xin Barag Zuoqi; XBY, Xin Barag Youqi

Animal sampling took place in Hulunbuir (47°05′–53°20′N, 115°31′–126°04′E), northeastern Inner Mongolia of China (Fig. 1), which is the border area of China, Russia and Mongolia. The surveyed region spanned forest area of 120,000 km^2^ and grassland area of 80,000 km^2^ [6]. Sheep and cattle are the most common domesticated animals in this region, due to the abundant herbage resources. A total of 480 serum samples of sheep and cattle were collected in May 2017 for detection of viral RNA, specific antibodies, neutralizing antibodies and isolation of viruses. The sampling areas were selected due to the high incidence of human ALSV infection cases.

### Preparation of recombinant VP2 protein

Considering the capsid protein function of VP2 in Jingmenviruses, the recombinant VP2 protein of H3 strain (GenBank: MH158418) was expressed in *Escherichia coli* and purified for ALSV-specific antibodies detection by ELISA [1, 5]. Briefly, after codon optimization with MaxCodonTM Optimization Program V13 (DetaiBio, Nanjing, China), the VP2 sequence was synthesized by DetaiBio and cloned to vector pET30a by *Nde*I-*Hind*III clone site. The recombinant VP2 protein was then expressed in BL21 (DE3) *E. coli* and induced by 0.1 mM IPTG at 15 °C for 16 h. After collection, the bacteria were sonicated and purified using a Ni-IDA purification system (Detai Bio) according to the manufacturer’s protocol. Finally, the recombinant protein was confirmed by SDS-PAGE and Western blot.

### Enzyme-linked immunosorbent assay (ELISA)

ALSV-specific antibodies in sheep and cattle were detected by using an indirect ELISA as described elsewhere [7]. Briefly, recombinant VP2 protein was used as the coating antigen with 0.2 μg/well for 96-well plates; after being coated overnight and blocked with 5% skim milk-PBS, 50 μl of 10-fold diluted serum samples were added to the plates and incubated at 37 °C for 1 h. After washing three times, the plates were added 50 μl of horseradish peroxidase (HRP)-conjugated rabbit anti-sheep or anti-cattle IgG antibodies (1:20000, Abcam). Fifty microliters of TMB peroxidase substrate [3,3′,5,5′ tetramethylbenzidine and hydrogen peroxide (H_2_O_2_)] was used for color development after washing five times; the optical density (OD) was measured by using a spectrometer (ELx800; BioTek, Winooski, USA) at 450 nm. Each sample was tested in triplicate. The cut-off value of the reaction was calculated as the mean optical density of ten ALSV-negative control sera plus 3 standard deviations. Based on the formula described before [ELISA index (EI) = OD sample/OD cut-off value] a sample with EI values ≥ 1.2 was considered positive [8]. Sheep or cattle ALSV ELISA positive serum samples (1:10) were two-fold serially diluted with a start of 1:10 to 1:320 to evaluate the ELISA titer of ALSV antibodies.

### Viral neutralization test (VNT)

A microneutralization assay was conducted to evaluate viral neutralizing antibodies as previously described [9]. Sheep or cattle ALSV ELISA positive serum samples were 2-fold serially diluted with a start of 1:10, and mixed with an equal volume of 100 median tissue culture infectious doses (TCID_50_) of ALSV (strain H3), followed by incubation at 37 °C for 1.5 h. The mixture was added to cultivated Vero cells in a 96-well plate in quadruplicate. The plates were then incubated at 37 °C with 5% CO_2_ for 7 days. Viral infection was evaluated by an immunofluorescence assay (IFA). After fixation with 4% phosphate-buffered paraformaldehyde for 1 h, the plates were blocked with 5% bovine serum albumin (BSA) at room temperature for 1 h and treated with 1% Triton X-100 for 15 min. They were then washed with PBS and incubated with 50-fold diluted convalescent-phase ALSV patient sera for 1 h. Finally, the plates were incubated with Alexa Fluor 488-labeled goat anti-human IgG antibodies. The end-point titer was shown as the reciprocal of the highest dilution of the serum, at which the number of the foci was < 50%.

### Quantitative real-time RT-PCR

Total RNA of all serum samples was extracted using a RNA Extraction kit (MiniBEST Viral RNA/DNA Extraction Kit Ver.5.0; TaKaRa, Shiga, Japan) according to manufacturer’s instructions and stored at −80 °C for use. Quantitative real-time RT-PCR (RT-qPCR) was conducted on RNA of serum samples by using a SYBR Green Supermix kit (TaKaRa) according to the manufacturer’s instructions. The primers used in the system included the forward primer (5′-GGC TAA ACA CAT CAA ACA-3′) and the reverse primer (5′-GCA TCC AGG TCA TAG TTA-3′), which targeted the ALSV NS3 gene. The cut-off cycle threshold (Ct) value was set at 35 cycles for a positive reaction, and the sample was considered positive if it had a Ct value below the cut-off. A standard curve from positive control sample was used to determine the viral RNA copy numbers of the positive samples.

### Virus isolation and sequence analysis

The confirmed ALSV positive samples were inoculated into Vero cell line for virus isolation as described previously [1]. After 3 passages, viral RNA was extracted from the Vero cells for ALSV detection. The partial S1 and S3 segments of isolated ALSV was sequenced using Sanger sequencing (Comate Bioscience, Changchun, China). The sequenced nucleotide of the ALSV isolates from sheep and cattle were compared with the ALSV isolates from ticks, patients and other Jingmenviruses, and analyzed using the Maximum Likelihood method (ML) with Tamura-Nei model and a bootstrap test of 1000 replicates to confirm the results.

### Statistical analyses

The rates and 95% confidence intervals of viral RNA and specific antibodies in sheep and cattle were calculated by the binomial proportion method, and statistically analyzed by either the Chi-square test or Fisher’s exact test using SAS software v.9.3 (SAS Institute Inc., Cary, NC, USA). Viral RNA copies in animals were analyzed by the Kruskal-Wallis test using GraphPad Prism v.5.0 (GraphPad Software, Inc., San Diego, CA, USA). Values of *P *< 0.05 were regarded as statistically significant.

## Results

### Serological detection of ALSV infection

The recombinant VP2 protein was successfully expressed with a size of 27 kDa, and confirmed with human ALSV convalescent phase serum by using Western blot (Additional file 1: Figure S1). We sampled 240 sheep and 240 cattle in Hulunbuir to evaluate the prevalence of ALSV-specific antibodies by VP2-ELISA and VNT. The prevalence of viral ELISA antibodies was significantly higher (*χ*^2^ = 3.94, *df* = 1, *P* = 0.047) in sheep (22/240, 9.2%, 95% CI: 5.8–13.6%) than that in cattle (11/240, 4.6%, 95% CI: 2.3–8.1%) (Table 1, Additional file 2: Tables S1, S2). The antibody titre of positive sera measured by ELISA showed a highest titer of 1:160 in sheep with a geometric mean titer (GMT) of 1:27, and a highest titer of 1:80 in cattle with a GMT of 1:21 (Additional file 2: Table S3). All of the ELISA-positive samples were tested by virus neutralization test (VNT), but no significant difference was found between sheep (10/240, 4.2%, 95% CI: 2.02–7.53%) and cattle (4/240, 1.7%, 95% CI: 0.46–4.21%) (*χ*^2^ = 1.84, *df* = 1, *P* = 0.18), with a geometric mean titer (GMT) of 1:23 in sheep and 1:20 in cattle, respectively (Table 1, Additional file 2: Tables S1–S3). A high prevalence was detected in sheep in Genhe (4/60, 6.7%, 95% CI: 1.9–16.2%) and Oroqen (3/60, 5.0%, 95% CI: 1.0–13.9%) by VNT, while a low prevalence was found in sheep from Eʼerguna (2/60, 3.3%, 95% CI: 0.4–11.5%) and Yakeshi (1/30, 3.3%, 95% CI: 0.1–17.2%), and in cattle in Genhe (2/60, 3.3%, 95% CI: 0.4–11.5%), Moqi (1/30, 3.3%, 95% CI: 0.1–17.2%) and Yakeshi (1/30, 3.3%, 95% CI: 0.1–17.2%) (Table 1). No VNT-positive samples were detected in sheep in Moqi, and in cattle in Eʼerguna and Oroqen.

**Table 1:** Prevalence of ALSV in sheep and cattle in Hulunbuir, Inner Mongolia of China

Animal	Region	ELISA prevalence (%)	VNT prevalence (%)	RT-qPCR prevalence (%)
Sheep	Genhe	13.3 (8/60)	6.7 (4/60)	31.7 (19/60)
	E’erguna	6.7 (4/60)	3.3 (2/60)	40.0 (24/60)
	Oroqen	10.0 (6/60)	5.0 (3/60)	15.2 (5/60)
	Moqi	6.7 (2/30)	0.0 (0/30)	20.0 (6/30)
	Yakeshi	6.7 (2/30)	3.3 (1/30)	30.0 (9/30)
	Total	9.2 (22/240)	4.2 (10/240)	26.3 (63/240)
Cattle	Genhe	8.3 (5/60)	3.3 (2/60)	33.3 (20/60)
	E’erguna	1.7 (1/60)	0.0 (0/60)	31.7 (19/60)
	Oroqen	3.3 (2/60)	0.0 (0/60)	23.3 (14/60)
	Moqi	6.7 (2/30)	3.3 (1/30)	20.0 (6/30)
	Yakeshi	3.3 (1/30)	3.3 (1/30)	23.3 (7/30)
	Total	4.6 (11/240)	1.7 (4/240)	27.5 (66/240)

*Abbreviations*: ELISA, enzyme-linked immunosorbent assay; VNT, viral neutralization test; RT-qPCR, quantitative real-time reverse transcription polymerase chain reaction

### Molecular detection of ALSV infection

Molecular detection of ALSV infection in sheep and cattle was conducted by RT-qPCR, showing a prevalence of 26.3% (63/240, 95% CI: 20.8–32.3%) in sheep and 27.5% (66/240, 95% CI: 21.85–33.15%) in cattle, and no significant difference was found between the two animal species (*χ*^2^ = 0.10, *df* = 1, *P* = 0.76). The mean (± standard deviation, SD) of the logarithmic transformation of ALSV RNA load levels was 3.74 ± 0.34 and 3.87 ± 0.58 log_10_ RNA copies/ml in sheep and cattle, respectively, within 3–5 log_10_ RNA copies/ml for most animals (95%; Additional file 1: Figure S2; Additional file 2: Tables S1, S2). Of the 63 sheep and 66 cattle serum samples tested positive by RT-qPCR, only 23.8% (15/63, 95% CI: 13.98–36.21%) were ELISA-positive and 12.7% (8/63, 95% CI: 5.65–23.50%) tested VNT-positive in sheep, and 6.1% (4/66, 95% CI: 1.68–14.80%) were ELISA-positive and 4.6% (3/66, 95% CI: 0.95–12.71%) tested VNT-positive in cattle (Table 2).

**Table 2:** Comparative results of RT-qPCR with ELISA and VNT in sheep and cattle

RT-qPCR	ELISA			VNT		
	Positive	Negative	Total	Positive	Negative	Total
Sheep						
Positive	15	48	63	8	55	63
Negative	7	170	177	2	175	177
Total	22	218	240	10	230	240
Cattle						
Positive	4	62	66	3	63	66
Negative	7	167	174	1	173	174
Total	11	229	240	4	236	240

### Virus isolation and sequence analysis

**Fig. 2:**
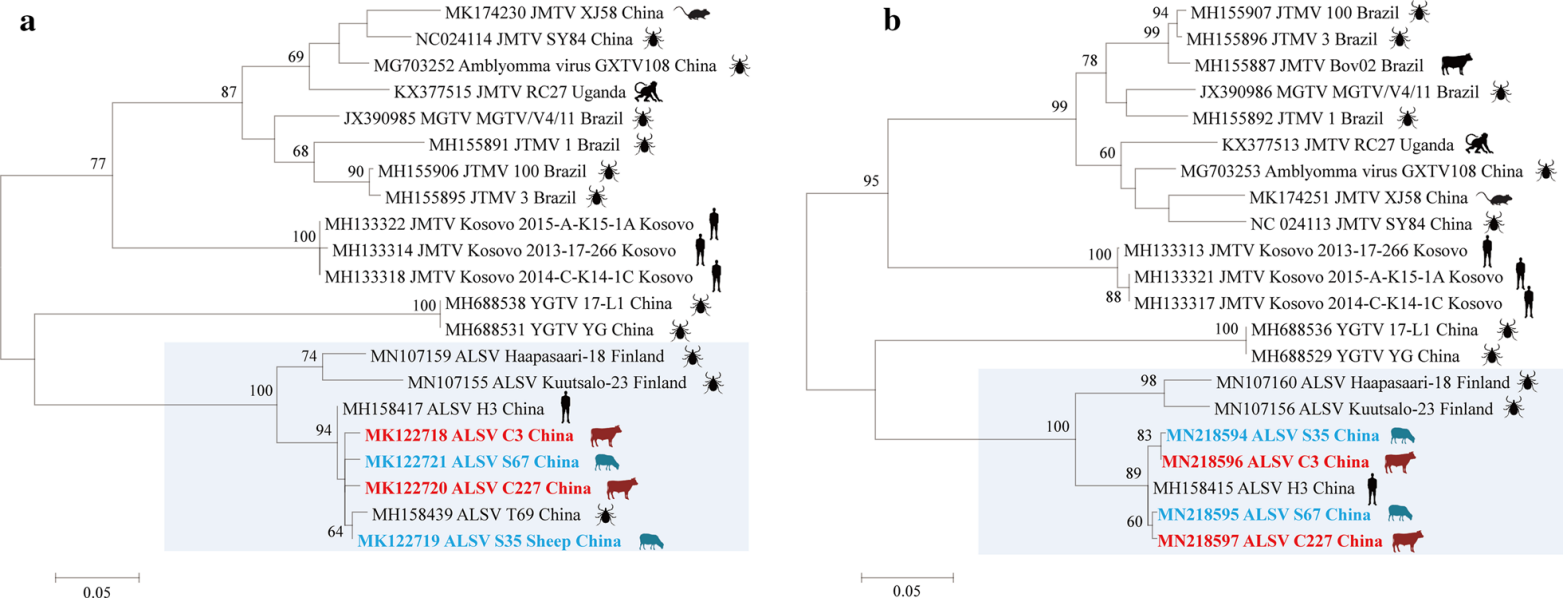
Phylogenetic analysis of partial segment S3 (**a**, NS3-like) and S1 (**b**, NS5-like) from isolated ALSVs from patients, ticks, cattle, sheep and other Jingmenviruses. Sequences are identified by their GenBank accession numbers, followed by the virus name, strain and country. All of the Jingmenviruses are also labeled with the isolate source with silhouette picture. The scale-bars in each panel indicate 0.05 substitutions per site. *Abbreviations*: ALSV, Alongshan virus; JMTV, Jingmen tick virus; MGTV, Mogiana tick virus; YGTV, Yanggou tick virus

Virus isolation was performed on all viral RNA-positive samples (*n* = 103), but ALSV was only successfully isolated from 2 sheep serum samples of Genhe and Eʼerguna (S35 and S67) and 2 cattle of Genhe and Yakeshi (C3 and C227), respectively (Additional file 1: Figures S3–S5). Phylogenetic analysis of ALSVs indicated that the sheep and cattle isolates were genetically close to ALSVs in patient (H3) and *I. persulcatus* tick (T69) in the studied areas (Fig. 2). All sequences of the isolates from domesticated animals, patients and ticks shared more than 98% identity, showing a close evolutionary relationship among those ALSV isolates from domesticated animals, ticks and ALSV patients.

## Discussion

From 480 serum samples collected from sheep and cattle in ALSV-endemic regions of Inner Mongolia, we provide evidence of the circulation and natural infection of ALSV in these animals. High positivity of ALSV RNA in collected sheep and cattle (26.3–27.5%) was found, while much lower seropositivity of ALSV-specific antibodies in sheep and cattle (VNT, 1.7–4.2%) was detected (Table 1). Moreover, the detection of RNA of ALSV from domesticated animals suggests ALSV viremia in sheep and cattle. These findings and previous studies indicate that ALSV infection may be an emerging tick-borne zoonosis.

The transmission of an arbovirus must include an infective virus, a competent vector and susceptible hosts. The sheep and cattle in our studied regions grazed on pastures, and were heavily infested with ticks from May through July. *Ixodes persulcatus* has been considered as a potential vector of ALSV, which is the important tick species in these regions. ALSV RNA has previously been detected in *I. persulcatus* ticks collected from grass by using RT-PCR, with a prevalence of 6.5% in disease-endemic areas [1]. Some viral isolates have been obtained from ticks in a previous study, and from sheep and cattle in the present study from the same regions. Phylogenetic analysis of the virus isolates from sheep and cattle in this study and from *I. persulcatus* ticks in our previous study showed > 98% homology with ALSV isolates from patients in the same area, which suggests a potential link of ALSV infections among ticks, domesticated animals, and humans.

ALSV is a novel member of the Jingmenvirus group in the family *Flaviviridae*, which is more closely related to Jingmen tick virus (JMTV) with a nucleotide identity of 57.4–74.5%. JMTV was first isolated from *Rhipicephalus microplus* in central and eastern China, and also detected in a wide range of hosts, including cattle, dogs and goats [5]. Meanwhile, a variant named Mogiana tick virus was isolated from *R. microplus* and cattle in Brazil [3, 10–12], and a RC27 variant isolated from a red colobus monkey in Uganda [4].

Previous studies have shown that JMTV in cattle has a high seroprevalence of 37.2% in China and a low molecular prevalence of 9.0% and 14.0% in China and Brazil, respectively [5, 12]. In contrast, the present study showed a low prevalence by ELISA (4.6–9.2%) and VNT (1.2–4.7%), but a high prevalence by RT-qPCR (26.3–27.5%) of ALSV in sheep and cattle. The possible reasons that the seropositivity is higher than that of RNA positivity are as follows. First, the majority of the sheep and cattle groups we collected were young animals (data not shown), and most of them were less than one year-old. Combined with the epidemic season of ticks from May to July every year, this indicates that many animals experienced the ticks for the first time. Secondly, we collected the serum samples in May; this is the beginning of the season when cattle and sheep are grazing and ticks are active, in which period cattle and sheep were newly infected with ALSV and formed viremia, but the body had not yet produced antibodies for humoral immune response. Finally, it is also possible that ALSV itself has some immune escape mechanism, and lead to persistent infections in cattle and sheep. As evidenced in previous studies, JMTV is not easy to isolate in many cell lines like C6/36, BME26 and DH82 cells [5, 12]. However, in the present study, we isolated the ALSV successfully using Vero cells with a slightly cytopathic effect, which may be due to the low nucleotide identity of 57.4–74.5% and difference structure of VP1 (Envelope protein) between ALSV and JMTV [1]. Although RNA and antibodies to JMTV were detected in cattle in China and Brazil, no JMTV-related symptoms were described in the studies [5, 12]. In the present study, no symptoms were observed in cattle and sheep when they were sampled, but it does not mean ALSV is non-pathogenic to cattle and sheep. More epidemiological surveys should be performed on grazing animals on different season at the epidemic place, and experimental infection of herding animals with ALSV should be performed to collect data on antibody production and presence of viremia.

This study has several limitations. First, only four viral isolates were isolated which limits the ability to understand viral diversity. Secondly, ALSV infections in ticks from animals were not investigated; thus, it is difficult to confirm the direct association of ALSV infection in ticks, animals and human patients. Thirdly, it is not possible to confirm that ALSV is pathogenic to the studied animals in this study.

## Conclusions

Our findings show that ALSV is circulating among sheep and cattle in the disease-endemic areas of China. These animals have high infection rates of ALSV, and may serve as amplifying hosts in the virus epidemic season. The natural transmission model of ALSV should be further elucidated, including the duration of viremia, the possibility of persistent infection in animals, potential reservoirs, and links of ALSV infections among animals and humans, which may contribute to formulate effective measures for controlling this emerging tick-borne zoonosis.

## Supplementary information

**Additional file 1: Figure S1.** Expression and identification of recombinant VP2 protein of ALSV in *Escherichia coli*. **a** The purified recombinant VP2 protein analyzed by SDS-PAGE. Lane 1: 2 μg sampling amount of BSA; Lane 2: molecular protein marker; Lane 3: purified recombinant VP2 protein with 2 μg sampling amount. **b** Western blot analysis of the recombinant VP2 protein. Lane 1: molecular protein marker; Lane 2: expressed VP2 protein. **Figure S2.** RNA load of ALSV in sheep and cattle in Hulunbuir, northeastern Inner Mongolia of China. **Figure S3.** ALSV-induced cellular changes (cytopathic effect, CPE) in Vero cells. **a** CPE in Vero cells with ALSV infection. **b** Control Vero cells without virus infection. **Figure S4.** ALSV isolated from sheep and cattle in Vero cells detected by an immunofluorescence assay (IFA). **a** Virus grown in Vero cells detected by IFA using the serum sample of a ALSV patient. **b** Control of IFA using the serum sample without ALSV infection. **Figure S5.** Nested RT-PCR results of first to third passages supernatant of ALSV strain C3 from cattle isolated by Vero cells. Lines 1–3: cell culture supernatant from first to third passages; Line 4: cell culture supernatant of Vero cells without ALSV infection; Line M: DNA marker DL2000 from Takara.

**Additional file 2: Table S1.** ELISA, VNT and RT-qPCR detection results in sheep in Hulunbuir, Inner Mongolia of China. **Table S2.** ELISA, VNT and RT-qPCR detection results in cattle in Hulunbuir, Inner Mongolia of China. **Table S3.** Antibody titre of ALSV positive sera of sheep and cattle measured by ELISA and VNT, separately.

## Data Availability

All data generated or analyzed in this study are included in this article and its additional files.

## Abbreviations

ALSV: Alongshan virus
ELISA: enzyme-linked immunosorbent assay
VNT: viral neutralization test
RT-qPCR: quantitative real-time reverse transcription polymerase chain reaction
JMTV: Jingmen tick virus
IFA: immunofluorescence assay
GMT: geometric mean titer
