# Supplementary material for: Prevalence of the emerging novel Alongshan virus infection in sheep and cattle in Inner Mongolia, northeastern China
Source: Parasit Vectors. 2019 Sep 12;12:450. doi: 10.1186/s13071-019-3707-1 (PMC6740026; doi:10.1186/s13071-019-3707-1)
Supplement: Supplementary file 1 — Additional file 1: Figure S1. Expression and identification of recombinant VP2 protein of ALSV in Escherichia coli. a The purified recombinant VP2 protein analyzed by SDS-PAGE. Lane 1: 2 μg sampling amount of BSA; Lane 2: molecular protein marker; Lane 3: purified recombinant VP2 protein with 2 μg sampling amount. b Western blot analysis of the recombinant VP2 protein. Lane 1: molecular protein marker; Lane 2: expressed VP2 protein. Figure S2. RNA load of ALSV in sheep and cattle in Hulunbuir, northeastern Inner Mongolia of China. Figure S3. ALSV-induced cellular changes (cytopathic effect, CPE) in Vero cells. a CPE in Vero cells with ALSV infection. b Control Vero cells without virus infection. Figure S4. ALSV isolated from sheep and cattle in Vero cells detected by an immunofluorescence assay (IFA). a Virus grown in Vero cells detected by IFA using the serum sample of a ALSV patient. b Control of IFA using the serum sample without ALSV infection. Figure S5. Nested RT-PCR results of first to third passages supernatant of ALSV strain C3 from cattle isolated by Vero cells. Lines 1–3: cell culture supernatant from first to third passages; Line 4: cell culture supernatant of Vero cells without ALSV infection; Line M: DNA marker DL2000 from Takara. [file 13071_2019_3707_MOESM1_ESM.pdf]

Additional file 1

Figure S1. Expression and identification of recombinant VP2 protein of ALSV in *Escherichia coli*.

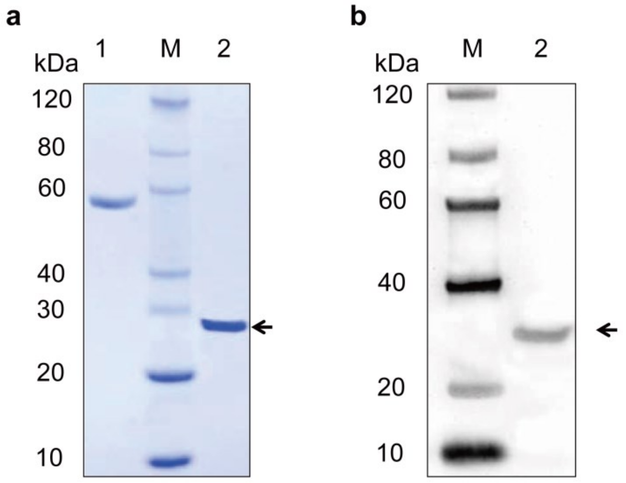

(**a**) The purified recombinant VP2 protein analyzed by SDS-PAGE. Lane 1: 2 μg sampling amount of BSA; Lane 2: Molecular protein marker; Lane 3: The purified recombinant VP2 protein with 2 μg sampling amount. (**b**) Western blot analysis of the recombinant VP2 protein. Lane 1: Molecular protein marker; Lane 2: The expressed VP2 protein.

Figure S2. RNA load of ALSV in sheep and cattle in Hulunbuir, northeastern Inner Mongolia of China.

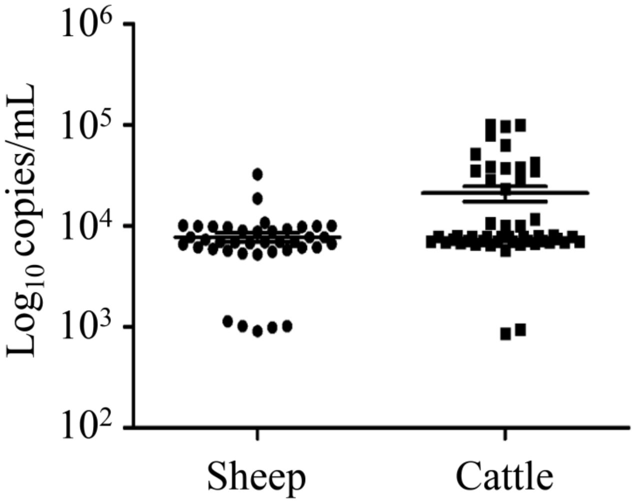

Figure S3. ALSV-induced cellular changes (cytopathic effect, CPE) in Vero cells.

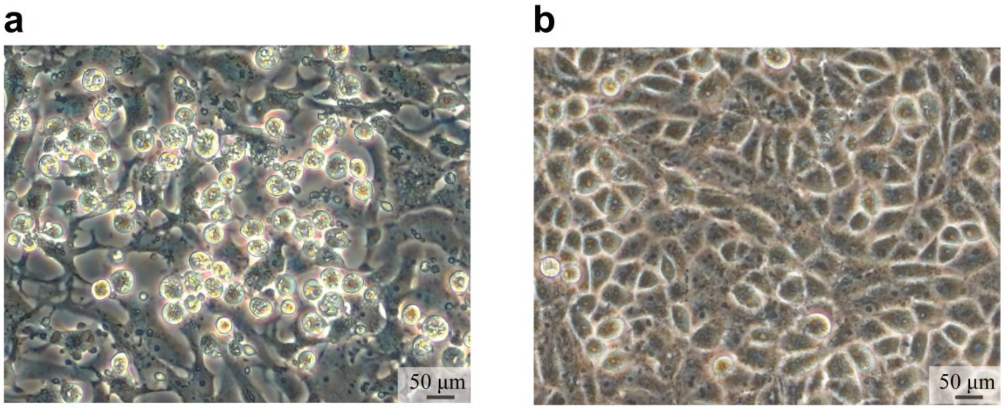

Panel **a** shows CPE in Vero cells with ALSV infection. Panel **b** shows the control Vero cells without virus infection.

Figure S4. ALSV isolated from sheep and cattle in Vero cells detected by an immunofluorescence assay (IFA)

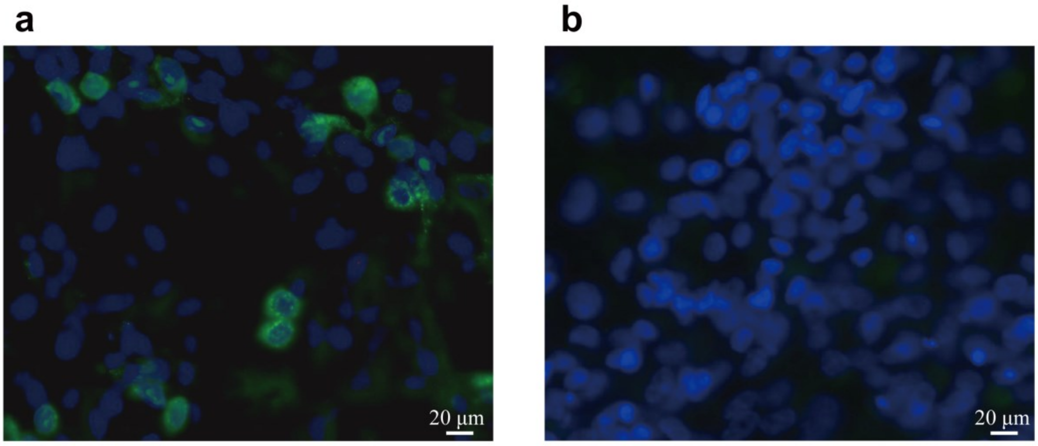

Panel **a** shows the virus grown in Vero cells detected by IFA using the serum sample of a ALSV patient. Panel **b** shows the control of IFA using the serum sample without ALSV infection.

Figure S5. Nested RT-PCR results of first to third passages supernatant of ALSV strain C3 from cattle isolated by Vero cells.

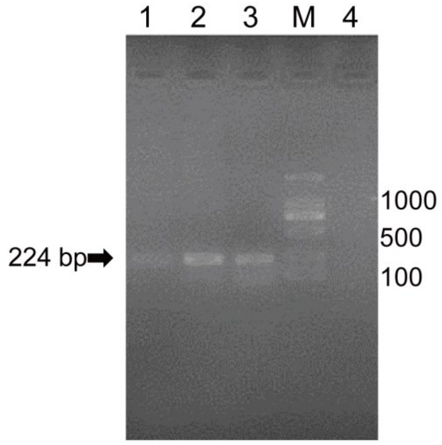

Lines 1-3: Cell culture supernatant from first to third passages; Line 4: Cell culture supernatant of Vero cells without ALSV infection; Line M: DNA marker DL2000 from Takara.
