# Supplementary material for: Prevalence of the emerging novel Alongshan virus infection in sheep and cattle in Inner Mongolia, northeastern China
Source: Parasit Vectors. 2019 Sep 12;12:450. doi: 10.1186/s13071-019-3707-1 (PMC6740026; doi:10.1186/s13071-019-3707-1)
Supplement: Supplementary file 2 — Additional file 2: Table S1. ELISA, VNT and RT-qPCR detection results in sheep in Hulunbuir, Inner Mongolia of China. Table S2. ELISA, VNT and RT-qPCR detection results in cattle in Hulunbuir, Inner Mongolia of China. Table S3. Antibody titre of ALSV positive sera of sheep and cattle measured by ELISA and VNT, separately. [file 13071_2019_3707_MOESM2_ESM.docx]

**Additional file 2**

**Table S1.** ELISA, VNT, and RT-qPCR detection results in sheep in Hulunbuir, Inner Mongolia of China.

| NO. | Region | EI | ELISA result | VNT titer | VNT result | RNA load copies/mL | RT-qPCR result |
| --- | --- | --- | --- | --- | --- | --- | --- |
| 1 | Genhe | 0.62 | - | NA | NA | - | - |
| 2 | Genhe | 0.41 | - | NA | NA | - | - |
| 3 | Genhe | 0.29 | - | NA | NA | - | - |
| 4 | Genhe | 0.32 | - | NA | NA | - | - |
| 5 | Genhe | 0.27 | - | NA | NA | - | - |
| 6 | Genhe | 0.41 | - | NA | NA | - | - |
| 7 | Genhe | 0.35 | - | NA | NA | - | - |
| 8 | Genhe | 1.44 | 10 | <20 | - | 908.32 | + |
| 9 | Genhe | 0.32 | - | NA | NA | 9954.11 | + |
| 10 | Genhe | 1.44 | 10 | <20 | - | 6962.16 | + |
| 11 | Genhe | 0.46 | - | NA | NA | - | - |
| 12 | Genhe | 0.28 | - | NA | NA | - | - |
| 13 | Genhe | 0.44 | - | NA | NA | 5764.13 | + |
| 14 | Genhe | 0.66 | - | NA | NA | - | - |
| 15 | Genhe | 0.43 | - | NA | NA | 10754.89 | + |
| 16 | Genhe | 0.33 | - | NA | NA | - | - |
| 17 | Genhe | 0.39 | - | NA | NA | 6132.77 | + |
| 18 | Genhe | 1.09 | - | NA | NA | - | - |
| 19 | Genhe | 0.43 | - | NA | NA | - | - |
| 20 | Genhe | 0.62 | - | NA | NA | - | - |
| 21 | Genhe | 0.46 | - | NA | NA | - | - |
| 22 | Genhe | 0.48 | - | NA | NA | - | - |
| 23 | Genhe | 1.14 | - | NA | NA | - | - |
| 24 | Genhe | 0.38 | - | NA | NA | 5992.1 | + |
| 25 | Genhe | 0.57 | - | NA | NA | - | - |
| 26 | Genhe | 0.37 | - | NA | NA | - | - |
| 27 | Genhe | 0.39 | - | NA | NA | - | - |
| 28 | Genhe | 0.52 | - | NA | NA | - | - |
| 29 | Genhe | 0.63 | - | NA | NA | 6562.14 | + |
| 30 | Genhe | 0.72 | - | NA | NA | 9187.21 | + |
| 31 | Genhe | 0.64 | - | NA | NA | - | - |
| 32 | Genhe | 0.84 | - | NA | NA | - | - |
| 33 | Genhe | 0.24 | - | NA | NA | 8993.65 | + |
| 34 | Genhe | 1.22 | 20 | 20 | + | 1136.98 | + |
| 35 | Genhe | 0.63 | - | NA | NA | 32435.8 | + |
| 36 | Genhe | 0.43 | - | NA | NA | - | - |
| 37 | Genhe | 0.82 | - | NA | NA | - | - |
| 38 | Genhe | 0.77 | - | NA | NA | - | - |
| 39 | Genhe | 1.23 | 40 | 20 | + | - | - |
| 40 | Genhe | 0.55 | - | NA | NA | - | - |
| 41 | Genhe | 0.58 | - | NA | NA | - | - |
| 42 | Genhe | 0.51 | - | NA | NA | - | - |
| 43 | Genhe | 0.38 | - | NA | NA | - | - |
| 44 | Genhe | 0.51 | - | NA | NA | - | - |
| 45 | Genhe | 0.79 | - | NA | NA | 9794.7 | + |
| 46 | Genhe | 1.66 | 40 | <20 | - | 7752.19 | + |
| 47 | Genhe | 1.30 | 10 | <20 | - | - | - |
| 48 | Genhe | 0.92 | - | NA | NA | - | - |
| 49 | Genhe | 0.68 | - | NA | NA | - | - |
| 50 | Genhe | 0.63 | - | NA | NA | - | - |
| 51 | Genhe | 0.86 | - | NA | NA | - | - |
| 52 | Genhe | 1.02 | - | NA | NA | 6972.3 | + |
| 53 | Genhe | 1.17 | - | NA | NA | 7722.88 | + |
| 54 | Genhe | 0.95 | - | NA | NA | 5216.67 | + |
| 55 | Genhe | 1.32 | 80 | 40 | + | 5894.45 | + |
| 56 | Genhe | 0.58 | - | NA | NA | - | - |
| 57 | Genhe | 0.54 | - | NA | NA | - | - |
| 58 | Genhe | 0.95 | - | NA | NA | - | - |
| 59 | Genhe | 0.96 | - | NA | NA | - | - |
| 60 | Genhe | 1.20 | 40 | 20 | + | 18754.89 | + |
| 61 | E'erguna | 0.93 | - | NA | NA | 6894.45 | + |
| 62 | E'erguna | 0.74 | - | NA | NA | 6232.33 | + |
| 63 | E'erguna | 0.57 | - | NA | NA | - | - |
| 64 | E'erguna | 0.69 | - | NA | NA | - | - |
| 65 | E'erguna | 1.01 | - | NA | NA | - | - |
| 66 | E'erguna | 0.59 | - | NA | NA | - | - |
| 67 | E'erguna | 0.66 | - | NA | NA | 8876.11 | + |
| 68 | E'erguna | 1.14 | - | NA | NA | 6031.55 | + |
| 69 | E'erguna | 0.35 | - | NA | NA | - | - |
| 70 | E'erguna | 0.69 | - | NA | NA | - | - |
| 71 | E'erguna | 0.30 | - | NA | NA | - | - |
| 72 | E'erguna | 1.07 | - | NA | NA | 6099.4 | + |
| 73 | E'erguna | 0.95 | - | NA | NA | - | - |
| 74 | E'erguna | 0.93 | - | NA | NA | - | - |
| 75 | E'erguna | 1.07 | - | NA | NA | 6135.67 | + |
| 76 | E'erguna | 0.46 | - | NA | NA | 6471.33 | + |
| 77 | E'erguna | 0.94 | - | NA | NA | 6832.26 | + |
| 78 | E'erguna | 0.62 | - | NA | NA | - | - |
| 79 | E'erguna | 0.52 | - | NA | NA | - | - |
| 80 | E'erguna | 0.44 | - | NA | NA | - | - |
| 81 | E'erguna | 0.35 | - | NA | NA | 9763.74 | + |
| 82 | E'erguna | 0.65 | - | NA | NA | 5864.3 | + |
| 83 | E'erguna | 1.97 | 40 | <20 | - | 991.35 | + |
| 84 | E'erguna | 0.70 | - | NA | NA | 5534.47 | + |
| 85 | E'erguna | 0.88 | - | NA | NA | 6885.32 | + |
| 86 | E'erguna | 0.78 | - | NA | NA | - | - |
| 87 | E'erguna | 0.69 | - | NA | NA | 6922.42 | + |
| 88 | E'erguna | 0.62 | - | NA | NA | - | - |
| 89 | E'erguna | 0.39 | - | NA | NA | - | - |
| 90 | E'erguna | 0.49 | - | NA | NA | - | - |
| 91 | E'erguna | 1.36 | 20 | <20 | - | - | - |
| 92 | E'erguna | 0.87 | - | NA | NA | - | - |
| 93 | E'erguna | 1.17 | - | NA | NA | 9261.77 | + |
| 94 | E'erguna | 0.61 | - | NA | NA | - | - |
| 95 | E'erguna | 0.30 | - | NA | NA | - | - |
| 96 | E'erguna | 0.45 | - | NA | NA | 6176.31 | + |
| 97 | E'erguna | 0.43 | - | NA | NA | - | - |
| 98 | E'erguna | 0.88 | - | NA | NA | 6991.71 | + |
| 99 | E'erguna | 1.13 | - | NA | NA | - | - |
| 100 | E'erguna | 0.63 | - | NA | NA | - | - |
| 101 | E'erguna | 0.47 | - | NA | NA | - | - |
| 102 | E'erguna | 1.21 | 40 | 20 | + | 7326.54 | + |
| 103 | E'erguna | 2.70 | 160 | 20 | + | 1018.13 | + |
| 104 | E'erguna | 0.69 | - | NA | NA | - | - |
| 105 | E'erguna | 0.80 | - | NA | NA | 6831.4 | + |
| 106 | E'erguna | 0.47 | - | NA | NA | 9211.2 | + |
| 107 | E'erguna | 0.57 | - | NA | NA | - | - |
| 108 | E'erguna | 0.79 | - | NA | NA | - | - |
| 109 | E'erguna | 0.60 | - | NA | NA | - | - |
| 110 | E'erguna | 0.71 | - | NA | NA | - | - |
| 111 | E'erguna | 0.72 | - | NA | NA | 7631.7 | + |
| 112 | E'erguna | 0.94 | - | NA | NA | - | - |
| 113 | E'erguna | 0.73 | - | NA | NA | - | - |
| 114 | E'erguna | 0.60 | - | NA | NA | - | - |
| 115 | E'erguna | 0.84 | - | NA | NA | - | - |
| 116 | E'erguna | 0.49 | - | NA | NA | 1323.8 | + |
| 117 | E'erguna | 0.60 | - | NA | NA | - | - |
| 118 | E'erguna | 0.63 | - | NA | NA | 989.43 | + |
| 119 | E'erguna | 0.49 | - | NA | NA | - | - |
| 120 | E'erguna | 0.60 | - | NA | NA | - | - |
| 121 | Oroqen | 0.27 | - | NA | NA | - | - |
| 122 | Oroqen | 0.30 | - | NA | NA | - | - |
| 123 | Oroqen | 0.27 | - | NA | NA | 5568.96 | + |
| 124 | Oroqen | 0.19 | - | NA | NA | - | - |
| 125 | Oroqen | 0.38 | - | NA | NA | - | - |
| 126 | Oroqen | 0.28 | - | NA | NA | - | - |
| 127 | Oroqen | 0.46 | - | NA | NA | - | - |
| 128 | Oroqen | 0.27 | - | NA | NA | - | - |
| 129 | Oroqen | 0.42 | - | NA | NA | - | - |
| 130 | Oroqen | 0.92 | - | NA | NA | - | - |
| 131 | Oroqen | 0.89 | - | NA | NA | - | - |
| 132 | Oroqen | 0.49 | - | NA | NA | - | - |
| 133 | Oroqen | 2.49 | 80 | 20 | + | - | - |
| 134 | Oroqen | 0.53 | - | NA | NA | - | - |
| 135 | Oroqen | 0.41 | - | NA | NA | - | - |
| 136 | Oroqen | 1.12 | - | NA | NA | - | - |
| 137 | Oroqen | 0.98 | - | NA | NA | - | - |
| 138 | Oroqen | 1.11 | - | NA | NA | - | - |
| 139 | Oroqen | 0.92 | - | NA | NA | 9991.63 | + |
| 140 | Oroqen | 0.82 | - | NA | NA | - | - |
| 141 | Oroqen | 2.23 | 40 | 20 | + | 5676.64 | + |
| 142 | Oroqen | 1.06 | - | NA | NA | - | - |
| 143 | Oroqen | 1.07 | - | NA | NA | - | - |
| 144 | Oroqen | 0.52 | - | NA | NA | - | - |
| 145 | Oroqen | 0.72 | - | NA | NA | - | - |
| 146 | Oroqen | 0.70 | - | NA | NA | - | - |
| 147 | Oroqen | 0.89 | - | NA | NA | - | - |
| 148 | Oroqen | 1.71 | 10 | <20 | - | - | - |
| 149 | Oroqen | 0.68 | - | NA | NA | - | - |
| 150 | Oroqen | 1.67 | 20 | <20 | - | 1018.13 | + |
| 151 | Oroqen | 0.37 | - | NA | NA | - | - |
| 152 | Oroqen | 0.63 | - | NA | NA | - | - |
| 153 | Oroqen | 1.03 | - | NA | NA | - | - |
| 154 | Oroqen | 2.19 | 80 | 40 | + | 5336.04 | + |
| 155 | Oroqen | 1.66 | 20 | <20 | - | - | - |
| 156 | Oroqen | 0.21 | - | NA | NA | - | - |
| 157 | Oroqen | 0.33 | - | NA | NA | - | - |
| 158 | Oroqen | 0.25 | - | NA | NA | - | - |
| 159 | Oroqen | 0.44 | - | NA | NA | - | - |
| 160 | Oroqen | 0.49 | - | NA | NA | - | - |
| 161 | Oroqen | 0.54 | - | NA | NA | - | - |
| 162 | Oroqen | 0.39 | - | NA | NA | - | - |
| 163 | Oroqen | 0.24 | - | NA | NA | - | - |
| 164 | Oroqen | 0.47 | - | NA | NA | - | - |
| 165 | Oroqen | 0.92 | - | NA | NA | - | - |
| 166 | Oroqen | 0.41 | - | NA | NA | - | - |
| 167 | Oroqen | 0.42 | - | NA | NA | - | - |
| 168 | Oroqen | 1.12 | - | NA | NA | - | - |
| 169 | Oroqen | 1.06 | - | NA | NA | - | - |
| 170 | Oroqen | 0.43 | - | NA | NA | - | - |
| 171 | Oroqen | 0.79 | - | NA | NA | - | - |
| 172 | Oroqen | 0.35 | - | NA | NA | - | - |
| 173 | Oroqen | 0.37 | - | NA | NA | - | - |
| 174 | Oroqen | 0.60 | - | NA | NA | - | - |
| 175 | Oroqen | 0.46 | - | NA | NA | - | - |
| 176 | Oroqen | 1.00 | - | NA | NA | - | - |
| 177 | Oroqen | 0.63 | - | NA | NA | - | - |
| 178 | Oroqen | 0.64 | - | NA | NA | - | - |
| 179 | Oroqen | 0.24 | - | NA | NA | - | - |
| 180 | Oroqen | 0.70 | - | NA | NA | - | - |
| 181 | Moqi | 1.09 | - | NA | NA | - | - |
| 182 | Moqi | 1.17 | - | NA | NA | - | - |
| 183 | Moqi | 0.73 | - | NA | NA | - | - |
| 184 | Moqi | 0.52 | - | NA | NA | - | - |
| 185 | Moqi | 1.20 | 10 | <20 | - | - | - |
| 186 | Moqi | 0.50 | - | NA | NA | 1223.21 | + |
| 187 | Moqi | 0.48 | - | NA | NA | - | - |
| 188 | Moqi | 0.98 | - | NA | NA | - | - |
| 189 | Moqi | 0.35 | - | NA | NA | - | - |
| 190 | Moqi | 0.48 | - | NA | NA | 954.23 | - |
| 191 | Moqi | 0.81 | - | NA | NA | 10071.11 | + |
| 192 | Moqi | 0.52 | - | NA | NA | - | - |
| 193 | Moqi | 0.86 | - | NA | NA | - | - |
| 194 | Moqi | 0.26 | - | NA | NA | - | - |
| 195 | Moqi | 0.78 | - | NA | NA | - | - |
| 196 | Moqi | 1.09 | - | NA | NA | - | - |
| 197 | Moqi | 0.66 | - | NA | NA | - | - |
| 198 | Moqi | 0.84 | - | NA | NA | - | - |
| 199 | Moqi | 0.94 | - | NA | NA | - | - |
| 200 | Moqi | 0.74 | - | NA | NA | - | - |
| 201 | Moqi | 0.50 | - | NA | NA | 6638.29 | + |
| 202 | Moqi | 1.53 | 10 | <20 | - | 6092.37 | + |
| 203 | Moqi | 1.13 | - | NA | NA | - | - |
| 204 | Moqi | 0.34 | - | NA | NA | - | - |
| 205 | Moqi | 0.17 | - | NA | NA | 8812.39 | + |
| 206 | Moqi | 0.34 | - | NA | NA | - | - |
| 207 | Moqi | 0.86 | - | NA | NA | - | - |
| 208 | Moqi | 0.62 | - | NA | NA | - | - |
| 209 | Moqi | 0.95 | - | NA | NA | - | - |
| 210 | Moqi | 0.36 | - | NA | NA | - | - |
| 211 | Yakeshi | 0.41 | - | NA | NA | - | - |
| 212 | Yakeshi | 0.78 | - | NA | NA | - | - |
| 213 | Yakeshi | 0.39 | - | NA | NA | 6753.01 | + |
| 214 | Yakeshi | 0.70 | - | NA | NA | - | - |
| 215 | Yakeshi | 0.44 | - | NA | NA | - | - |
| 216 | Yakeshi | 1.34 | 40 | 20 | + | 7742.88 | + |
| 217 | Yakeshi | 0.34 | - | NA | NA | - | - |
| 218 | Yakeshi | 0.42 | - | NA | NA | 5243.27 | + |
| 219 | Yakeshi | 0.38 | - | NA | NA | 9871.66 | + |
| 220 | Yakeshi | 0.89 | - | NA | NA | - | - |
| 221 | Yakeshi | 0.14 | - | NA | NA | - | - |
| 222 | Yakeshi | 0.39 | - | NA | NA | - | - |
| 223 | Yakeshi | 0.37 | - | NA | NA | - | - |
| 224 | Yakeshi | 0.29 | - | NA | NA | 7742.88 | + |
| 225 | Yakeshi | 0.73 | - | NA | NA | - | - |
| 226 | Yakeshi | 0.68 | - | NA | NA | - | - |
| 227 | Yakeshi | 0.25 | - | NA | NA | - | - |
| 228 | Yakeshi | 0.38 | - | NA | NA | - | - |
| 229 | Yakeshi | 0.30 | - | NA | NA | - | - |
| 230 | Yakeshi | 0.35 | - | NA | NA | 998.8 | + |
| 231 | Yakeshi | 0.55 | - | NA | NA | 5853.7 | + |
| 232 | Yakeshi | 0.39 | - | NA | NA | - | - |
| 233 | Yakeshi | 0.51 | - | NA | NA | - | - |
| 234 | Yakeshi | 0.96 | - | NA | NA | - | - |
| 235 | Yakeshi | 0.92 | - | NA | NA | - | - |
| 236 | Yakeshi | 0.44 | - | NA | NA | 6938.73 | + |
| 237 | Yakeshi | 0.80 | - | NA | NA | - | - |
| 238 | Yakeshi | 0.65 | - | NA | NA | - | - |
| 239 | Yakeshi | 1.04 | - | NA | NA | - | - |
| 240 | Yakeshi | 1.28 | 10 | <20 | - | 10138.8 | + |

**Table S2.** ELISA, VNT, and RT-qPCR detection results in cattle in Hulunbuir, Inner Mongolia of China.

| NO. | Region | EI | ELISA result | VNT titer | VNT result | RNA load copies/mL | RT-qPCR result |
| --- | --- | --- | --- | --- | --- | --- | --- |
| 1 | Genhe | 1.03 | - | NA | NA | 10018.37 | + |
| 2 | Genhe | 0.75 | - | NA | NA | - | - |
| 3 | Genhe | 0.82 | - | NA | NA | 99801.54 | + |
| 4 | Genhe | 0.63 | - | NA | NA | - | - |
| 5 | Genhe | 1.60 | 20 | <20 | - | - | - |
| 6 | Genhe | 0.97 | - | NA | NA | - | - |
| 7 | Genhe | 0.92 | - | NA | NA | - | - |
| 8 | Genhe | 1.15 | - | NA | NA | 904.13 | + |
| 9 | Genhe | 0.91 | - | NA | NA | - | - |
| 10 | Genhe | 1.45 | 10 | <20 | - | - | - |
| 11 | Genhe | 0.64 | - | NA | NA | 11701.5 | + |
| 12 | Genhe | 1.05 | - | NA | NA | - | - |
| 13 | Genhe | 0.83 | - | NA | NA | - | - |
| 14 | Genhe | 0.67 | - | NA | NA | - | - |
| 15 | Genhe | 0.73 | - | NA | NA | 6632.17 | + |
| 16 | Genhe | 0.96 | - | NA | NA | - | - |
| 17 | Genhe | 0.89 | - | NA | NA | 10715.07 | + |
| 18 | Genhe | 0.79 | - | NA | NA | - | - |
| 19 | Genhe | 0.67 | - | NA | NA | - | - |
| 20 | Genhe | 0.98 | - | NA | NA | - | - |
| 21 | Genhe | 0.76 | - | NA | NA | - | - |
| 22 | Genhe | 0.67 | - | NA | NA | - | - |
| 23 | Genhe | 0.83 | - | NA | NA | 6988.61 | + |
| 24 | Genhe | 0.82 | - | NA | NA | - | - |
| 25 | Genhe | 0.77 | - | NA | NA | 7764.79 | + |
| 26 | Genhe | 0.99 | - | NA | NA | - | - |
| 27 | Genhe | 0.64 | - | NA | NA | 8084.33 | + |
| 28 | Genhe | 0.78 | - | NA | NA | 7089.55 | + |
| 29 | Genhe | 0.91 | - | NA | NA | - | - |
| 30 | Genhe | 0.82 | - | NA | NA | - | - |
| 31 | Genhe | 0.64 | - | NA | NA | - | - |
| 32 | Genhe | 1.07 | - | NA | NA | - | - |
| 33 | Genhe | 1.48 | 10 | <20 | - | 6704.8 | + |
| 34 | Genhe | 0.77 | - | NA | NA | 10035.1 | + |
| 35 | Genhe | 0.49 | - | NA | NA | 51384.79 | + |
| 36 | Genhe | 0.95 | - | NA | NA | - | - |
| 37 | Genhe | 0.74 | - | NA | NA | - | - |
| 38 | Genhe | 0.74 | - | NA | NA | - | - |
| 39 | Genhe | 0.79 | - | NA | NA | - | - |
| 40 | Genhe | 0.49 | - | NA | NA | - | - |
| 41 | Genhe | 1.36 | 40 | 20 | + | 7089.55 | + |
| 42 | Genhe | 0.66 | - | NA | NA | - | - |
| 43 | Genhe | 0.90 | - | NA | NA | - | - |
| 44 | Genhe | 0.99 | - | NA | NA | - | - |
| 45 | Genhe | 0.88 | - | NA | NA | 7018.6 | + |
| 46 | Genhe | 0.99 | - | NA | NA | - | - |
| 47 | Genhe | 0.67 | - | NA | NA | - | - |
| 48 | Genhe | 0.75 | - | NA | NA | - | - |
| 49 | Genhe | 0.60 | - | NA | NA | - | - |
| 50 | Genhe | 0.44 | - | NA | NA | - | - |
| 51 | Genhe | 0.92 | - | NA | NA | 42435.8 | + |
| 52 | Genhe | 0.62 | - | NA | NA | - | - |
| 53 | Genhe | 0.98 | - | NA | NA | 7326.45 | + |
| 54 | Genhe | 0.59 | - | NA | NA | - | - |
| 55 | Genhe | 0.62 | - | NA | NA | 7076.33 | + |
| 56 | Genhe | 0.86 | - | NA | NA | - | - |
| 57 | Genhe | 0.62 | - | NA | NA | - | - |
| 58 | Genhe | 1.66 | 80 | 20 | + | 936.16 | + |
| 59 | Genhe | 0.96 | - | NA | NA | 7874.31 | + |
| 60 | Genhe | 1.03 | - | NA | NA | - | - |
| 61 | E'erguna | 0.75 | - | NA | NA | - | - |
| 62 | E'erguna | 0.62 | - | NA | NA | - | - |
| 63 | E'erguna | 0.96 | - | NA | NA | - | - |
| 64 | E'erguna | 0.76 | - | NA | NA | - | - |
| 65 | E'erguna | 0.63 | - | NA | NA | - | - |
| 66 | E'erguna | 0.54 | - | NA | NA | - | - |
| 67 | E'erguna | 0.76 | - | NA | NA | - | - |
| 68 | E'erguna | 0.68 | - | NA | NA | - | - |
| 69 | E'erguna | 0.81 | - | NA | NA | - | - |
| 70 | E'erguna | 0.97 | - | NA | NA | - | - |
| 71 | E'erguna | 0.80 | - | NA | NA | - | - |
| 72 | E'erguna | 0.77 | - | NA | NA | - | - |
| 73 | E'erguna | 0.70 | - | NA | NA | - | - |
| 74 | E'erguna | 0.90 | - | NA | NA | - | - |
| 75 | E'erguna | 0.50 | - | NA | NA | - | - |
| 76 | E'erguna | 0.53 | - | NA | NA | - | - |
| 77 | E'erguna | 0.51 | - | NA | NA | 916.38 | + |
| 78 | E'erguna | 0.62 | - | NA | NA | - | - |
| 79 | E'erguna | 0.59 | - | NA | NA | - | - |
| 80 | E'erguna | 0.56 | - | NA | NA | - | - |
| 81 | E'erguna | 0.74 | - | NA | NA | - | - |
| 82 | E'erguna | 0.65 | - | NA | NA | - | - |
| 83 | E'erguna | 0.83 | - | NA | NA | - | - |
| 84 | E'erguna | 0.68 | - | NA | NA | - | - |
| 85 | E'erguna | 0.74 | - | NA | NA | 8041.37 | + |
| 86 | E'erguna | 0.65 | - | NA | NA | - | - |
| 87 | E'erguna | 0.59 | - | NA | NA | 987.8 | + |
| 88 | E'erguna | 0.58 | - | NA | NA | 966.52 | + |
| 89 | E'erguna | 0.63 | - | NA | NA | - | - |
| 90 | E'erguna | 0.67 | - | NA | NA | 987.5 | + |
| 91 | E'erguna | 0.53 | - | NA | NA | - | - |
| 92 | E'erguna | 0.60 | - | NA | NA | - | - |
| 93 | E'erguna | 0.62 | - | NA | NA | - | - |
| 94 | E'erguna | 0.65 | - | NA | NA | 943.67 | + |
| 95 | E'erguna | 0.85 | - | NA | NA | 7756.12 | + |
| 96 | E'erguna | 0.65 | - | NA | NA | - | - |
| 97 | E'erguna | 0.83 | - | NA | NA | - | - |
| 98 | E'erguna | 0.49 | - | NA | NA | 971.7 | + |
| 99 | E'erguna | 0.80 | - | NA | NA | - | - |
| 100 | E'erguna | 0.47 | - | NA | NA | - | - |
| 101 | E'erguna | 0.82 | - | NA | NA | 9835.31 | + |
| 102 | E'erguna | 0.97 | - | NA | NA | 79382.09 | + |
| 103 | E'erguna | 0.86 | - | NA | NA | 913.31 | + |
| 104 | E'erguna | 0.64 | - | NA | NA | - | - |
| 105 | E'erguna | 0.57 | - | NA | NA | 933.4 | + |
| 106 | E'erguna | 1.32 | 20 | <20 | - | - | - |
| 107 | E'erguna | 0.93 | - | NA | NA | - | - |
| 108 | E'erguna | 0.77 | - | NA | NA | 99801.54 | + |
| 109 | E'erguna | 0.74 | - | NA | NA | - | - |
| 110 | E'erguna | 0.63 | - | NA | NA | - | - |
| 111 | E'erguna | 0.66 | - | NA | NA | - | - |
| 112 | E'erguna | 0.61 | - | NA | NA | 96765.67 | + |
| 113 | E'erguna | 0.81 | - | NA | NA | - | - |
| 114 | E'erguna | 0.68 | - | NA | NA | - | - |
| 115 | E'erguna | 0.71 | - | NA | NA | 63352.41 | + |
| 116 | E'erguna | 0.72 | - | NA | NA | 963.17 | + |
| 117 | E'erguna | 0.87 | - | NA | NA | 6891.74 | + |
| 118 | E'erguna | 0.64 | - | NA | NA | - | - |
| 119 | E'erguna | 0.71 | - | NA | NA | 985.91 | + |
| 120 | E'erguna | 0.67 | - | NA | NA | 7834.8 | + |
| 121 | Oroqen | 0.84 | - | NA | NA | 23383.1 | + |
| 122 | Oroqen | 0.79 | - | NA | NA | - | - |
| 123 | Oroqen | 1.08 | - | NA | NA | - | - |
| 124 | Oroqen | 1.17 | - | NA | NA | 29709.27 | + |
| 125 | Oroqen | 0.92 | - | NA | NA | - | - |
| 126 | Oroqen | 0.55 | - | NA | NA | - | - |
| 127 | Oroqen | 0.66 | - | NA | NA | - | - |
| 128 | Oroqen | 0.54 | - | NA | NA | - | - |
| 129 | Oroqen | 0.91 | - | NA | NA | - | - |
| 130 | Oroqen | 0.99 | - | NA | NA | - | - |
| 131 | Oroqen | 0.80 | - | NA | NA | - | - |
| 132 | Oroqen | 0.73 | - | NA | NA | 7799.81 | + |
| 133 | Oroqen | 0.59 | - | NA | NA | - | - |
| 134 | Oroqen | 0.83 | - | NA | NA | - | - |
| 135 | Oroqen | 0.88 | - | NA | NA | 28403.11 | + |
| 136 | Oroqen | 0.76 | - | NA | NA | - | - |
| 137 | Oroqen | 0.83 | - | NA | NA | - | - |
| 138 | Oroqen | 1.20 | 10 | <20 | - | - | - |
| 139 | Oroqen | 0.83 | - | NA | NA | - | - |
| 140 | Oroqen | 0.55 | - | NA | NA | - | - |
| 141 | Oroqen | 0.78 | - | NA | NA | - | - |
| 142 | Oroqen | 0.85 | - | NA | NA | - | - |
| 143 | Oroqen | 0.95 | - | NA | NA | - | - |
| 144 | Oroqen | 0.67 | - | NA | NA | - | - |
| 145 | Oroqen | 0.72 | - | NA | NA | 35309.16 | + |
| 146 | Oroqen | 0.79 | - | NA | NA | - | - |
| 147 | Oroqen | 0.46 | - | NA | NA | 7175.21 | + |
| 148 | Oroqen | 1.43 | 10 | <20 | - | - | - |
| 149 | Oroqen | 0.53 | - | NA | NA | - | - |
| 150 | Oroqen | 0.92 | - | NA | NA | - | - |
| 151 | Oroqen | 0.39 | - | NA | NA | 6938.87 | + |
| 152 | Oroqen | 0.35 | - | NA | NA | - | - |
| 153 | Oroqen | 0.47 | - | NA | NA | 6527.6 | + |
| 154 | Oroqen | 0.45 | - | NA | NA | - | - |
| 155 | Oroqen | 0.45 | - | NA | NA | 7973.74 | + |
| 156 | Oroqen | 0.41 | - | NA | NA | - | - |
| 157 | Oroqen | 0.45 | - | NA | NA | - | - |
| 158 | Oroqen | 0.42 | - | NA | NA | - | - |
| 159 | Oroqen | 0.47 | - | NA | NA | - | - |
| 160 | Oroqen | 0.62 | - | NA | NA | - | - |
| 161 | Oroqen | 0.55 | - | NA | NA | - | - |
| 162 | Oroqen | 0.51 | - | NA | NA | 37605.81 | + |
| 163 | Oroqen | 0.43 | - | NA | NA | - | - |
| 164 | Oroqen | 0.37 | - | NA | NA | - | - |
| 165 | Oroqen | 0.41 | - | NA | NA | - | - |
| 166 | Oroqen | 0.62 | - | NA | NA | - | - |
| 167 | Oroqen | 0.41 | - | NA | NA | - | - |
| 168 | Oroqen | 0.40 | - | NA | NA | - | - |
| 169 | Oroqen | 0.38 | - | NA | NA | - | - |
| 170 | Oroqen | 0.39 | - | NA | NA | - | - |
| 171 | Oroqen | 0.39 | - | NA | NA | 7881.32 | + |
| 172 | Oroqen | 0.39 | - | NA | NA | - | - |
| 173 | Oroqen | 0.65 | - | NA | NA | 37732.66 | + |
| 174 | Oroqen | 0.44 | - | NA | NA | - | - |
| 175 | Oroqen | 0.39 | - | NA | NA | 7057.82 | + |
| 176 | Oroqen | 0.45 | - | NA | NA | - | - |
| 177 | Oroqen | 0.92 | - | NA | NA | 7881.32 | + |
| 178 | Oroqen | 0.55 | - | NA | NA | - | - |
| 179 | Oroqen | 0.66 | - | NA | NA | - | - |
| 180 | Oroqen | 0.54 | - | NA | NA | - | - |
| 181 | Moqi | 0.97 | - | NA | NA | - | - |
| 182 | Moqi | 0.78 | - | NA | NA | 6636.65 | + |
| 183 | Moqi | 0.88 | - | NA | NA | 899.42 | + |
| 184 | Moqi | 1.47 | 20 | <20 | - | - | - |
| 185 | Moqi | 0.71 | - | NA | NA | - | - |
| 186 | Moqi | 0.88 | - | NA | NA | - | - |
| 187 | Moqi | 0.90 | - | NA | NA | - | - |
| 188 | Moqi | 1.01 | - | NA | NA | 7889.05 | + |
| 189 | Moqi | 1.31 | 40 | 20 | + | 853.35 | + |
| 190 | Moqi | 1.01 | - | NA | NA | - | - |
| 191 | Moqi | 0.68 | - | NA | NA | - | - |
| 192 | Moqi | 0.78 | - | NA | NA | - | - |
| 193 | Moqi | 0.99 | - | NA | NA | - | - |
| 194 | Moqi | 0.90 | - | NA | NA | - | - |
| 195 | Moqi | 0.83 | - | NA | NA | - | - |
| 196 | Moqi | 0.85 | - | NA | NA | - | - |
| 197 | Moqi | 1.15 | - | NA | NA | - | - |
| 198 | Moqi | 0.91 | - | NA | NA | - | - |
| 199 | Moqi | 0.88 | - | NA | NA | - | - |
| 200 | Moqi | 0.98 | - | NA | NA | 6761.33 | + |
| 201 | Moqi | 0.67 | - | NA | NA | - | - |
| 202 | Moqi | 0.44 | - | NA | NA | - | - |
| 203 | Moqi | 0.85 | - | NA | NA | - | - |
| 204 | Moqi | 0.79 | - | NA | NA | - | - |
| 205 | Moqi | 0.55 | - | NA | NA | - | - |
| 206 | Moqi | 0.35 | - | NA | NA | - | - |
| 207 | Moqi | 0.41 | - | NA | NA | - | - |
| 208 | Moqi | 0.75 | - | NA | NA | - | - |
| 209 | Moqi | 0.92 | - | NA | NA | - | - |
| 210 | Moqi | 0.82 | - | NA | NA | 6905.9 | + |
| 211 | Yakeshi | 0.58 | - | NA | NA | 7467.11 | + |
| 212 | Yakeshi | 0.35 | - | NA | NA | - | - |
| 213 | Yakeshi | 0.45 | - | NA | NA | 7865.6 | + |
| 214 | Yakeshi | 0.53 | - | NA | NA | - | - |
| 215 | Yakeshi | 0.55 | - | NA | NA | 7812.43 | + |
| 216 | Yakeshi | 0.47 | - | NA | NA | - | - |
| 217 | Yakeshi | 0.54 | - | NA | NA | - | - |
| 218 | Yakeshi | 0.65 | - | NA | NA | - | - |
| 219 | Yakeshi | 0.68 | - | NA | NA | 35301.33 | + |
| 220 | Yakeshi | 0.51 | - | NA | NA | - | - |
| 221 | Yakeshi | 0.56 | - | NA | NA | 5751.77 | + |
| 222 | Yakeshi | 0.45 | - | NA | NA | - | - |
| 223 | Yakeshi | 0.63 | - | NA | NA | - | - |
| 224 | Yakeshi | 0.54 | - | NA | NA | - | - |
| 225 | Yakeshi | 0.47 | - | NA | NA | 7175.21 | + |
| 226 | Yakeshi | 0.57 | - | NA | NA | - | - |
| 227 | Yakeshi | 0.56 | - | NA | NA | 38317.42 | + |
| 228 | Yakeshi | 0.50 | - | NA | NA | - | - |
| 229 | Yakeshi | 0.58 | - | NA | NA | - | - |
| 230 | Yakeshi | 0.65 | - | NA | NA | - | - |
| 231 | Yakeshi | 0.71 | - | NA | NA | - | - |
| 232 | Yakeshi | 0.53 | - | NA | NA | - | - |
| 233 | Yakeshi | 0.54 | - | NA | NA | - | - |
| 234 | Yakeshi | 0.42 | - | NA | NA | - | - |
| 235 | Yakeshi | 0.55 | - | NA | NA | - | - |
| 236 | Yakeshi | 0.66 | - | NA | NA | - | - |
| 237 | Yakeshi | 0.61 | - | NA | NA | - | - |
| 238 | Yakeshi | 0.47 | - | NA | NA | - | - |
| 239 | Yakeshi | 1.78 | 40 | 20 | + | - | - |
| 240 | Yakeshi | 0.79 | - | NA | NA | - | - |

**Table S3.** Antibody titre of ALSV positive sera of sheep and cattle measured by ELISA and VNT, separately.

| Host | ELISA titer | | | | | | |  | VNT titer | | | |
| --- | --- | --- | --- | --- | --- | --- | --- | --- | --- | --- | --- | --- |
|  | 1:10 | 1:20 | 1:40 | 1:80 | 1:160 | Total | GMT |  | 1:20 | 1:40 | Total | GMT |
| Sheep | 7 | 4 | 7 | 3 | 1 | 22 | 1:27 |  | 8 | 2 | 10 | 1:23 |
| Cattle | 4 | 3 | 3 | 1 | 0 | 11 | 1:21 |  | 4 | 0 | 4 | 1:20 |

*Abbreviation*: GMT, geometrical mean titer.
